# Impact of Interactions between Melanoidins and Caffeine on the Bitter Taste of Coffee Beverages

**DOI:** 10.1021/acs.jafc.5c17022

**Published:** 2026-06-03

**Authors:** Michael Gigl, Johanna Kreissl, Oliver Frank

**Affiliations:** † Junior Research Group Food Processing and Health, ZIEL Institute for Food and Health, Technical University of Munich, Lise-Meitner-Str. 34, D-85354 Freising, Germany; ‡ Leibniz-Institute for Food Systems Biology at the Technical University of Munich, Lise-Meitner-Straße 34, 85354 Freising, Germany; § Chair of Food Chemistry and Molecular Sensory Science, TUM School of Life Sciences, Technical University of Munich, Lise-Meitner-Straße 34, 85354 Freising, Germany

**Keywords:** coffee, chlorogenic acid (CQA), caffeine, melanoidins, π–π interactions, NMR, complex formation

## Abstract

Since the discovery of caffeine in 1819 by Runge, its role in the sensory perception of coffee remains unclear. To further investigate its influence, the caffeine–chlorogenate complex was examined using NMR spectroscopy as well as sensory experiments. It was found that the complex formation between caffeine and chlorogenic acid (CQA) has no impact on the bitter taste of caffeine. The changes of the ^1^H NMR resonance signals of caffeine in the coffee matrix compared to an aqueous reference solution suggested that caffeine interacts, besides its complex with CQA, with the high molecular weight melanoidins containing fraction of coffee. After isolating the melanoidins from the coffee beverage, these interactions were confirmed in model studies using ^1^H NMR spectroscopy. Sensory studies with caffeine and the melanoidins in their natural concentrations showed a strong reduction in bitter intensity by about half compared to an aqueous caffeine solution of the same concentration.

## Graphical abstract

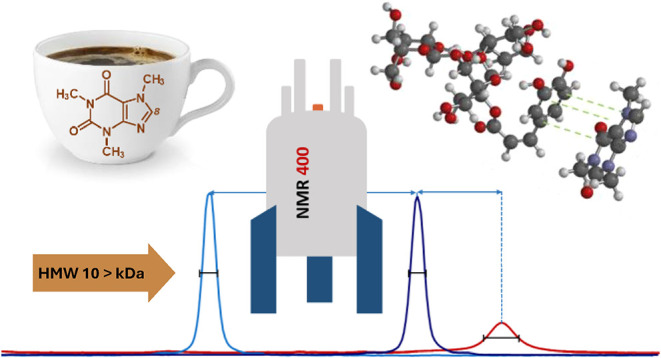

## Introduction

Freshly brewed coffee is appreciated by consumers all over the world, because of its stimulating effect, its attractive aroma, and its characteristic taste profile centring on sourness and pleasant bitterness. Flavor research performed within the last 30 years has shown that only a few out of several hundred volatile compounds contribute to the overall flavor of a freshly prepared coffee beverage. Sensory-based screenings of odorants by means of gas chromatography/olfactometry followed by their quantitative determination in combination with aroma reconstitution and omission experiments have impressively demonstrated that not much more than 30 odorants are required to mimic the typical aroma profile of a coffee brew.^1–3^ In comparison, the knowledge on the compounds responsible for the typical bitter taste developed during the roasting process is far from comprehensive.

Thermally generated compounds like furfurylalcohol, 5-hydroxymethyl-2-furaldehyde (HMF), various pyrazines as well as 2,5-diketopiperazines (DKP) were suggested as potential bitter compounds in roasted coffee, but for none of these compounds a contribution to the overall bitter taste of coffee could be shown.^4–7^ By application of the sensomics approach in combination with LC-MS/MS and 1D/2D NMR studies on coffee beverages and coffee related model systems, it could be shown that coffee roasting induces the transesterification, epimerization and lactonization of nonbitter compounds. For example, 3-, 4- and 5-*O*-caffeoylqunic acids (CQAs), as well as 3,4-, 3,5- and 4,5-dicaffeoylquinic acids (di-CQAs) giving rise to a multiple of intensely bitter tasting mono- and dicaffeoyl quinides (CQL, di-CQL).^8–10^ In the later stages of roasting these quinides will be degraded to 4-vinylcatechol, which oligomerizes to another class of harsh and lingering bitter compounds, the so-called phenylindanes.^11^ Kreppenhofer et al. showed that a series of bitter tasting (furan-2-yl)­methylated benzene diols and triols, which were formed upon the acid catalyzed dehydration of furfurylalcohol by an electrophilic substitution with di- and trihydroxybenzenes during coffee roasting.^12^ Very recently, Gigl et al. identified bitter tasting reaction products of volatile Strecker aldehydes such as acetaldehyde, propanal, methylpropanal and nonvolatile coffee compounds like quinic acid (QA), quinic acid lactone (QL) or 5-*O*-caffeoylquinic acid (5-CQA) as the representative of 3-, 4- and 5-CQA in coffee beverages.^13^ These compounds were formed by acetalization at the cis-diol function of quinic acid moiety of QA, QL and CQA. Very recently, the bitter compound mozambioside and its degradation products were exclusively identified in Arabica coffee.^14,15^ In addition, Gao et al. showed that coffee constituents like 4-caffeoylquinic acid, 5-caffeoylquinic acid, and 2-O-β-d-glucopyranosyl-atractyligenin are able to suppress the overall bitter taste of a coffee beverage.^16^ Two years later, the same authors reported that another group of five compounds formed during coffee roasting — four peptides and 2-O-β-d-glycopyranosyl-atractyligenin — were able to enhance the bitter taste of the coffee brew.^17^

Although multiple taste active molecules have been identified as coffee bitter compounds, the sensory significance for coffee quality of one of the most abundant bitter compounds, caffeine, is still unclear. On the advice of Goethe in 1819, Runge performed the first sensory experiments with a beverage prepared from green coffee beans, and he excluded the presence of any bitter compound because of the disgustingly sweet taste of the brew.^18,19^ More than 40 years ago, investigations performed by Chen revealed that the alkaloids caffeine and trigonelline, already present in raw coffee beans, contribute to a maximum of 10–30 and 1%, respectively, to the overall bitterness of a coffee beverage.^20^ No other bitter compound in coffee has been identified that shows a concentration that exceeds its threshold value so drastically, and for this reason, caffeine should play an important role in the overall bitter taste of coffee beverages. In literature, a caffeine-chlorogenate complex is discussed, which is formed via π–π interactions.^21–26^ The influence on the formation of such a complex on taste is still unclear. In addition, interactions between caffeine and the high molecular weight fraction (HMW) of coffee might play a role, since polyphenols can bind to the HMW in the course of the roasting process and are therefore, also able to provide π-electrons for these interactions.^27–29^ Gigl et al. demonstrated that certain aroma compounds, particularly 2-furfurylthiol (FFT) and pyrazines, can interact with the high molecular weight fraction of coffee, e.g., by formation of π–π interactions.^30,31^ Therefore, the aim of the present study was to clarify the real contribution of caffeine to the overall bitterness of coffee beverages on a molecular level and to show possible interactions of the alkaloid with other coffee constituents, which might influence the bitter taste, by using modern NMR techniques in combination with sensory-guided experiments.

## Material and Methods

### Chemicals

The following chemicals were obtained from Sigma-Aldrich (Steinheim, Germany): acetonitrile (≥99.9%), caffeic acid (≥99.0%), caffeine, 5-*O*-caffeoylquinic acid (5-CQA, ≥95%), deuterium oxide (D_2_O with TSP, 99.9 atom % D, 0.05% 3-(trimethylsilyl)­propionic-2,2,3,3-d_4_ acid sodium salt), formic acid (≥95%), hydrochloric acid (4 mol/L), ortho-phosphoric acid (85%), potassium chloride (≥99.0%), potassium dihydrogen phosphate (for analysis), potassium hydroxide (90%), sodium azide (p.a., ≥99.0%), sodium chloride (≥99.0%), sodium hydroxide (4 mol/L), trigonelline hydrochloride (analytical standard), 3-(trimethylsilyl)­propionic-2,2,3,3-d_4_ acid sodium salt (TSP, 98 atom % D).

Water (ultrapure) used for the NMR titration studies was purified with a Milli-Q Gradient A10 system (Millipore, Schwalbach, Germany). For sensory studies, bottled water (Evian, Danone, Wiesbaden, Germany) was used.

### Coffee Samples

Decaffeinated coffee beverages (5.6 g, capsa, *Espresso Decaffeinato*, Dallmayr, Munich, Germany) (manufacturer information: premium Arabica blend with a typical espresso roast, intensity 6 of 12.) were prepared with the respective capsule in a capsule coffee machine (Citiz, Krups, Frankfurt am Main, Germany). Each beverage was brewed with one capsule and 104 mL bottled water, immediately cooled to room temperature, and the missing volume was added to obtain a ratio of 5.4 g coffee powder/100 mL water.^1^ For the comparative sensory experiments, the decaffeinated coffee was aliquoted and caffeine was added to one part to obtain a beverage with a final concentration of 7.5 mmol/L.^32^ For the preparation of high molecular weight (HMW) fractions, the sum of five capsules were combined and ultrafiltrated. For the raw decaffeinated coffee beverage, one aliquot of the decaffeinated beans (Arabica from Colombia obtained from the coffee industry) were frozen with liquid nitrogen and ground in a grinder (analysis mill A11, basic, IKA, Staufen, Germany). The obtained raw coffee powder was used for preparing the raw decaffeinated coffee beverage. Therefore, raw coffee powder (54 g) was put in a coffee filter (Melitta Type 4, Minden, Germany), percolated (at 97 °C), immediately cooled down to room temperature, spiked with caffeine (7.5 mmol/L) and filled up to a final volume of 1 L with bottled water. Another aliquot of the same decaffeinated coffee beans was roasted at 240 °C for 4 min using a Probat BRZ II-type batch roaster (Emmerich, Germany).^33^ The roasted coffee beverage (54 g coffee/L water) was prepared as described for the raw decaffeinated coffee beverage and aliquoted; caffeine (7.5 mmol/L) was added to one of the roasted aliquots. The three samples were then compared in a sensory test.

### NMR Buffer

Potassium dihydrogen phosphate (10.2 g) was dissolved in deuterium oxide (40 mL), and potassium hydroxide (1.5 g) was added. Afterward, the pH (5.5) value was adjusted using ortho-phosphoric acid (85%). TSP (50 mg) and sodium azide (5 mg) were weighed in a volumetric flask (50 mL); the buffer solution was added, and filled up to the calibration mark with deuterium oxide.

### Influence on the Chemical Shifts of Caffeine and 5-CQA in Equimolar Ratios at Different Concentrations

Five equimolar solutions of caffeine and 5-CQA with increasing concentrations (0.31, 0.63, 1.25, 2.5, and 5.0 mmol/L, each) were solved in ultrapure water. All solutions were diluted with 10% NMR coffee buffer and measured using a ^1^H NMR experiment (noesygppr1d). For better visualization and comparison of the chemical shift differences of the complex with the individual compounds, the spectra of 5-CQA and caffeine were combined into a single spectrum using the TopSpin software (version 3.6, Bruker, Rheinstetten, Germany).

### Influence on the Chemical Shifts of Caffeine and 5-CQA in Different Molar Ratios of Both Compounds

For these studies, two solutions, one caffeine and one 5-CQA solution, were prepared in deuterium oxide, both with a concentration of 5.0 mmol/L. A number of 13 solutions were then pipetted in the following ratios: 5-CQA solution + caffeine solution (v:v), 0:100, 5:95; 10:90, 20:80; 30:70, 40:60, 50:50, 60:40, 70:30, 80:20, 90:10, 95:5, and 100:0. Whereby ratio 0:100 contained only caffeine and vice versa ratio 100:0 contained only 5-CQA. All mixtures were diluted with 10% NMR coffee buffer and measured using ^1^H NMR experiments (Table S1).

### Caffeine-Alkali Metal-Chlorogenate Complex Studies

A caffeine 5-CQA solution with different concentrations of potassium or sodium, respectively, was analyzed. Overall, four samples each, containing different amounts of potassium or sodium (1, 10, 100, and 1000 mmol/L) and a constant amount of caffeine (2.5 mmol/L) and 5-CQA (2.5 mmol/L) were prepared in deuterium oxide. In addition, one solution was prepared without the alkali salts containing only caffeine and 5-CQA (2.5 mmol/L, each). No NMR coffee buffer was added to avoid an additional ion input. All samples were measured via ^1^H NMR experiments.

### Ultrafiltration and Purification of the High Molecular Weight Fraction (HMW) from Coffee Beverages

The high molecular weight fraction of commercially available coffee (Dallmayr Capsa, Espresso, decaffeinato) was isolated using a crossflow ultrafiltration system (Sartorius Stedim Biotech, Goettingen, Germany) equipped with a molecular weight cutoff (MWCO) filter membrane (10 kDa) and was used to separate the coffee beverages into a HMW fraction >10 kDa and a low molecular fraction LMW fraction ≤ 10 kDa, respectively. For purification purposes, the HMW fraction was flushed with bottled water (8 L) to remove all low molecular weight compounds present in the coffee brew. The purification process was monitored by means of NMR spectroscopy, recording ^1^H NMR spectra after every washing step (1 L). Washing was continued until no resonance signals of LMW compounds, present in the aromatic region (5.5–9.5 ppm) of the NMR spectrum, were detectable. The HMW fraction was then concentrated to a fourth of its original volume by removing the flushing reservoir and stored at −20 °C until use.

### Alkaline Hydrolysis of the HMW Fraction

According to the literature, an aliquot of the 4-fold concentrated HMW fraction isolated as described previously was adjusted to pH 13 with a NaOH solution (4 mol/L). The mixture was stirred at 60 °C for 30 min and then neutralized with hydrochloric acid (4 mol/L). Afterward, this mixture was separated again into a HMW fraction >10 kDa (HMW_hydlz_) as well as a LMW fraction ≤ 10 kDa (as described perviously), and the liberated compounds (caffeic acid, *p*-coumaric acid, ferulic acid, and 3,4-dimethoxycinnamic acid), contained in the LMW fraction, were monitored by means of RP-HPLC/UV–vis. The comparison of U*V*_max_, retention time, and cochromatography with the reference compounds verified the success of the alkaline treatment.^31^

### Determination of Hydroxycinnamic Acids in Hydrolyzed HMW Fractions via UHPLC-MS/MS

A Shimadzu Nexera X2 UHPLC (Shimadzu, Duisburg, Germany), consisting of two pumps (LC30AD), a degasser (DGU 20 A5R), an autosampler (SIL 30AC, maintained at 5 °C), a column oven (CTO 30A, set to 40 °C), and a controller (CBM-20A) was connected to a QTRAP 5500 mass spectrometer (Sciex, Darmstadt, Germany) operating in negative electrospray ionization mode (ESI^–^). The following ESI source parameters were used for hydroxycinnamic acid (HCA) quantitation: ion spray voltage, −4500 V; curtain gas, 35 psi; nebulizer gas, 55 psi; heater gas, 65 psi; and source temperature, 450 °C. Data acquisition was done using Analyst 1.6.2 (Sciex, Darmstadt, Germany). Chromatographic separation of caffeic acid, ferulic acid, *p*-coumaric acid, and 3,4-dimethoxycinnamic acid was performed on a Kinetex Phenyl Hexyl column (100 × 2.1 mm i.d., 1.7 μm, Phenomenex, Aschaffenburg, Germany) with a flow rate of 0.4 mL/min. A gradient of aqueous formic acid (1%) as eluent A and methanol (1% formic acid) as eluent B was used and started with isocratic elution at 1% B for 1 min, increased to 50% B within 6 min, to 100% B in 1 min, and kept at 100% B for 2 min. The quantitation of target compounds was performed in triplicate according to Gigl et al. via external matrix calibration.^31^

### Nuclear Magnetic Resonance Spectroscopy (NMR)

NMR spectroscopic experiments were conducted on an Avance III 400 MHz spectrometer (Bruker, Rheinstetten, Germany), equipped with a Z-gradient 5 mm multinuclear inverse probe (BBI) (Bruker, Rheinstetten, Germany) at 25 °C. For structure elucidation, all experiment like correlation spectroscopy (COSY), heteronuclear single-quantum coherence (HSQC) as well as the heteronuclear multiple-bond correlation (HMBC), were executed on an Avance III 500 MHz system equipped with a cryo-TCI probe (Bruker, Rheinstetten, Germany) at 25 °C. Aliquots (600 μL) of each sample were analyzed in NMR tubes (5 × 178 mm, USC tubes, Bruker, Faellanden, Switzerland). All samples were diluted with 10% NMR buffer solution before measurement. In order to suppress the large water signal in the aqueous samples, all ^1^H NMR spectra were acquired using a NOESYPRESAT (noesygppr1d) experiment. For the caffeine-alkali metal-chlorogenate complex studies, a PROTON (zg) experiment was used. ^1^H chemical shifts were referenced to 3-(trimethylsilyl)­propionate-2,2,3,3-d_4_ (TSP) signal at δ 0.00. Data processing and analysis was done using TopSpin software (version 3.6, 400 MHz; version 3.2, 500 MHz, Bruker, Rheinstetten, Germany). ^1^H qNMR (qHNMR) was performed at 25 °C using the Bruker AV III 400 MHz system. The samples were dissolved in H_2_O/Buffer, and aliquots (600 μL) of each sample were analyzed in NMR tubes (5 × 178 mm), and the spectra were acquired using a NOESYPRESAT (noesygppr1d) experiment. Details on the exact procedure can be found in the literature.^34^

### Calculation of Electrostatic Potential Surfaces

The electrostatic potential surfaces for the representative aromatic units (caffeine and caffeic acid) were plotted from density functional (DFT) calculations (B3LYP using 6–31G*) with Spartan (Wave function, Inc., Irvine, CA, USA).

### General Conditions and Sensory Panel Training

Eighteen panelists (8 females, 10 males; 23–31 years in age), who had given informed consent to partake in the present sensory experiments and had no history of known taste disorders attended weekly training sessions for at least two years to become accustomed to the sensory procedures used, and to evaluate aqueous reference solutions of tastants. For the panel training, aqueous reference solutions (pH 5.5) were used, including sucrose (50 mmol/L) for sweet, l-lactic acid (20 mmol/L) for sour, caffeine (1 mmol/L) for bitter, and tannin (0.001%) for astringent perception. Samples for sensory evaluations were prepared in bottled water (Evian, Danone, Wiesbaden, Germany), and the pH-value was adjusted to pH 5.5 with an aqueous solution of formic acid (1%), respectively. Sensory analyses were conducted in a sensory panel room with individual booths at 22–25 °C while panelists wore nose clamps to negate possible nasal and retro-nasal olfactory cues. Optical differences in the sample solutions were masked by red lighting in the sensory cabins, the use of amber glass bottles, and, for samples containing melanoidin, by the addition of odor- and tasteless caramel color. Prior to sensory analysis, all analyzed compounds were checked for purity by means of NMR spectroscopy.

### Taste Profile Analysis

The panellists were asked to rate the intensities of the taste impressions bitter, sour, astringent, and sweet in the coffee samples on a scale from 0 (not detectable) to 5 (strongly detectable). The taste profiles were determined in two independent sessions. The determined values were averaged, and the standard deviation was calculated for each descriptor. Statistical differences in the bitter taste intensities of the coffee samples (caffeinated roast coffee beverage, decaffeinated roast coffee beverage, and raw coffee beverage) were assessed via *t* test with a significance level of α = 0.05.

### Analyses of Bitter-Taste Intensity

An aliquot of the HMW solution (>10 kDa) was diluted in its original concentration with water (5 mL concentrated HMW solution/20 mL water) to evaluate the intrinsic bitterness of coffee melanoidins. To assess the influence of complexation on bitter taste intensity, a second HMW sample was diluted with bottled water and spiked with caffeine (7.5 mmol/L) and an isomolar amount of 5-CQA. As a reference, an aqueous caffeine solution (7.5 mmol/L) was used. All samples were adjusted to pH 5.5 and then sensorially analyzed. The sensory panel was asked to rate the intensity of the taste quality “bitter” of these solutions on a scale from 0 (not detectable) to 5 (strongly detectable) in comparison to the reference caffeine-solution with a bitter taste intensity set to 5. Statistical differences in the bitter taste intensities were assessed via a *t* test with a significance level of α = 0.05.

### Determination of a Break-through Concentration for Caffeine Contribution to Overall Coffee Bitterness

In a three-alternative forced-choice test (3-AFC), decaffeinated coffee and decaffeinated coffee, spiked with defined amounts of caffeine, were evaluated by the sensory panel regarding bitter taste. For this purpose, freshly brewed decaffeinated coffee was prepared from capsules. The reference coffee was left untreated, while the other capsules were percolated directly into a beaker with caffeine accurately weighed in, ensuring complete dissolution. This resulted in final caffeine concentrations of 7.5, 10.0, and 12.5 mmol/L, respectively. Triangular series were prepared, consisting of two vessels containing decaffeinated coffee, serving as the reference, and one vessel containing either 7.5, 10.0, or 12.5 mmol/L of caffeine dissolved in decaffeinated coffee. The series was presented in increasing concentrations with randomized sample arrangements. The panelists were asked to identify the deviating sample out of the three presented samples, and statistically significant differences were determined based on the literature.^35^

## Ethic Statement

The panelists were informed in advance in writing about the respective research question and possible risks of the study. The data collected were used exclusively for this study and only within the scope of this study. The results were evaluated anonymously so that no conclusions could be drawn about the individual participants. There was no obligation to participate at any time and participants could withdraw from the study at any time. Consent to data processing was voluntary and could be withdrawn at any time without giving reasons and without any disadvantages. Participants also had the right to request the correction or deletion of their data. However, once the data had been anonymized, it was no longer possible to assign it to a specific person and the data could no longer be viewed, corrected or deleted. Due to the risk assessment, pregnant employees were generally excluded from the sensory studies.

## Results and Discussion

### Sensory Analysis of Different Coffee Beverages

In order to estimate the role of caffeine in the overall bitterness of freshly prepared coffee beverages, a first set of sensory experiments was carried out. First of all, a coffee brew was prepared from ground green coffee with an added caffeine concentration of 7.5 mmol/L and the sensory panelists were asked to rate the taste attributes sweet, sour, astringent, and bitter on a 5-point scale from 0 (not detectable) to 5 (strong impression). The results, given in Figure 1, show that besides low intensities for sweet (0.5), sour (1.25), and astringency (1.0), no significant bitter taste was detectable, although the concentration of the caffeine was more than factor 10 above its bitter detection threshold (0.67 mmol/L).^36^ On the one hand, these findings confirmed the observations of Runge,^18^ but on the other hand, the question arises: what is the reason for this observation? Since coffee beverages and products are typically made from roasted beans, the following sensory experiment was conducted: a coffee beverage prepared from decaffeinated roasted beans was split into two aliquots, and one of these aliquots was spiked with the same caffeine concentration (7.5 mmol/L) as the coffee brew made from green beans and sensorially compared to the nonspiked sample. Once again, the panelists were asked to evaluate the sweet, sour, astringent, and bitter taste sensations. The sweet and the astringent taste impressions of both beverages were judged equally, whereas the sour taste was rated slightly lower in the coffee with the added caffeine. Surprisingly, there was no significant difference in the bitter taste between the decaffeinated sample (3.8) and the sample (4.0), where caffeine was added with a value of approximately 10-fold above its bitter detection threshold (Figure 1).^36^ In addition, the typical unpleasant medicinal bitter taste of caffeine could not be perceived in the coffee spiked with caffeine in comparison to a pure caffeine solution with the same concentration. Only at a caffeine concentration of 12.5 mmol/L, which does not occur naturally in coffee beverages, were the panelists able to perceive the added caffeine due to its typical unpleasant bitterness and the significant (*p* ≤ 0.05) increase in bitter intensity. This was demonstrated by 3-AFC tests with 7.5, 10.0, and 12.5 mmol/L of caffeine added to decaffeinated coffee beverages.

**Figure 1:**
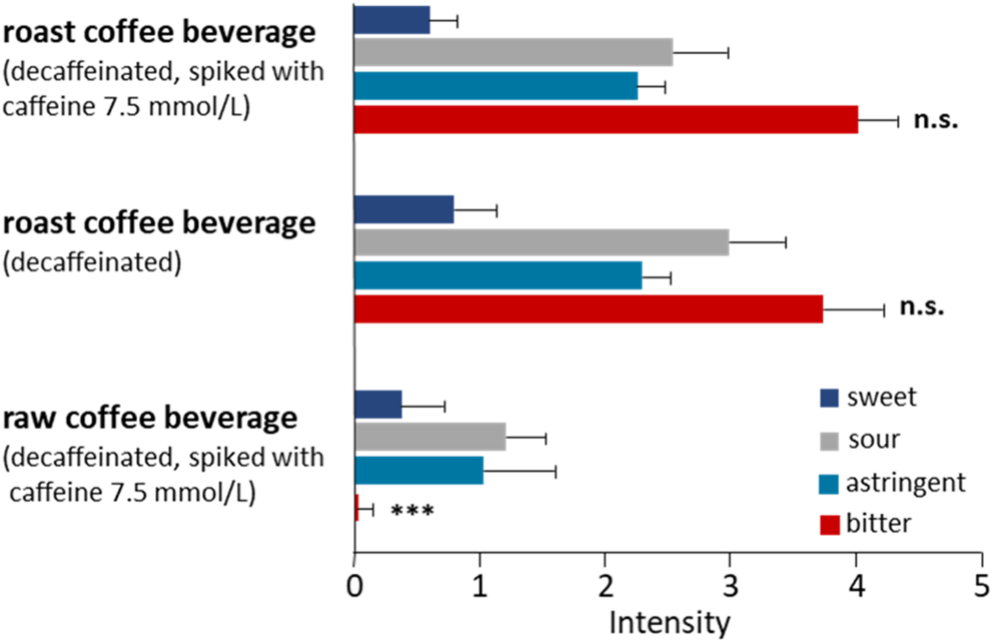
Sensory studies on bitter taste differences between a decaffeinated roasted and a decaffeinated roasted coffee beverage with added caffeine (7.5 mmol/L) as well as a raw coffee beverage with added caffeine (7.5 mmol/L). Statistical differences in bitter taste intensity were determined by *t* test (*** *p* ≤ 0.05, n.s. not significant).

There is evidence in the literature that could possibly explain these sensory results. Potassium-chlorogenate as a crystalline complex with caffeine was first isolated by Gorter and later characterized by Martin et al. using X-ray crystallographic analysis.^24,37^ In 1961, Sondheimer et al. described the so-called chlorogenic acid-caffeine complex in aqueous solution.^38^ The authors observed a bathochromic shift in the UV–vis spectra of the chlorogenic acid when caffeine was added and concluded that a charge-transfer complex was formed. About 10 years later, Horman and Viani examined this complex using NMR spectroscopy and came to the conclusion that the caffeine-chlorogenate complex could be described as a hydrophobically bound molecular π-complex.^22^ In 2009, D’Amelio et al. reinvestigated this complex by ^1^H and H,H NOESY NMR spectroscopy and suggested a different orientation of the aromatic rings compared to the results of Horman and Viani.^21,22^ In addition, beside the complex formation of caffeine with chlorogenic acid D’Amelio et al. and Nemzer et al. could also show complex formation of the three dicaffeoylquinic acid isomers with caffeine in model experiments as well as in caffeine-enriched whole coffee cherry extracts.^25,26^ Nevertheless, independent of the real orientation of the molecules within the complex, these groups were able to prove its existence in binary mixtures as well as in complex matrices like coffee cherry extracts or coffee beverages.^21,22,25,26^ Now the question arises: does this complex have an influence on the intrinsic bitter taste of caffeine, and are there any additional influencing factors in a complex matrix like coffee?

### ^1^H NMR of Caffeine, Caffeine Chlorogenate Complex, and a Freshly Prepared Coffee Beverage

Because X-ray crystallography is not suited to analyze molecular complexes like caffeine chlorogenate in their natural environment, high-resolution ^1^H NMR spectroscopy was the method of choice to analyze the behavior of caffeine in the coffee beverage as well as in the caffeine chlorogenate complex.^23,24^ First, a ^1^H NMR spectrum of a freshly prepared decaffeinated coffee beverage was recorded and compared with the same sample with the addition of 5 mmol/L caffeine (Figure 2). This concentration was chosen because, at this amount, caffeine is still sufficiently soluble to avoid cloudiness and the subsequent loss of quality in the NMR spectra. To prevent changes in the pH value as well as possible variations of the chemical shifts due to the addition of caffeine and to mimic the coffee as well as possible, the NMR sample was buffered at pH 5.5, which is a good average pH value for coffee beverages. As displayed in Figure 2, the chemical shifts of pH-sensitive compounds like formic acid, trigonelline, or *N*-methylpyridinium (NMP) were not affected by the addition of caffeine, indicating that there is no change in the pH value. In contrast, the signals resonating between 6.2 and 6.5, 6.7–7.2, and 7.5–7.7 ppm were strongly influenced by the presence of caffeine. These signals could be assigned to the hydroxycinnamic acid moieties of the chlorogenic- (CQA), feruloyl quinic acid (FQA) etc. isomers (H–C­(2′, 5′-8′)) by comparison with the commercially available 5-chlorogenic acid (5-CQA) as well as by recording 2D H–C correlated experiments like HMBC or HSQC and matching of the chemical shifts with literature data.^8,39,40^ All protons of the hydroxycinnamic acid moieties of the CQA, FQA derivatives in the decaffeinated coffee with added caffeine were more strongly shielded, i.e., shifted to lower frequencies, compared to the solution without caffeine, whereas all other signals within the aromatic region were unaffected (Figure 2). In contrast to previous publications, the direct influence of caffeine on the chemical shifts of hydroxycinnamic acid derivatives in coffee beverages and vice versa could be demonstrated, not only by comparing the binary complex (caffeine-chlorogenic acid) with coffee (espresso). Furthermore, unlike in the existing literature, all experiments were conducted at pH of 5.5 - typical for coffee - since the chemical shifts of NMR signals usually depend on the pH value.^21,22^

**Figure 2:**
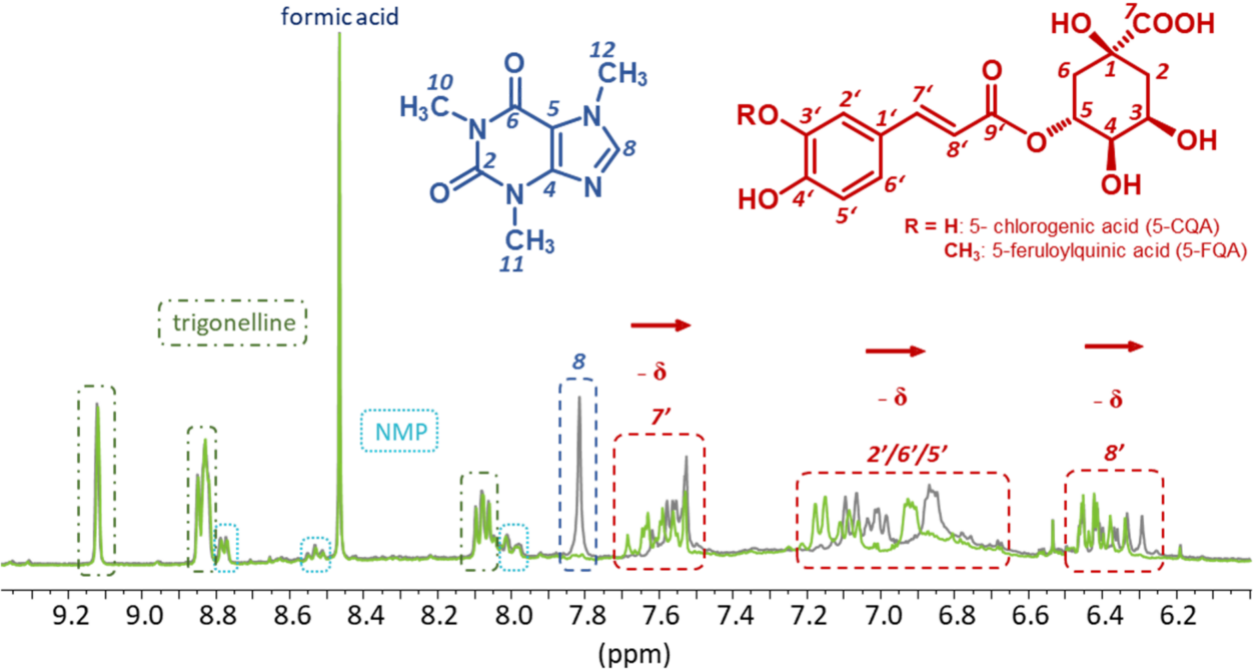
Excerpt of ^1^H NMR spectra (400 MHz, noesypresat, 540 μL sample, 60 μL NMR coffee buffer, 25 °C, pH = 5.5) of decaffeinated coffee beverage (green line) in comparison to decaffeinated coffee beverage spiked with caffeine (5 mmol/L) (gray line). The structural formula of 5-CQA is shown in the figure as an example. In addition to 5-CQA, 3- and 4-CQA and other hydroxycinnamic acid derivatives also occur naturally in coffee and therefore, also show resonances in this region of the ^1^H NMR spectra.

Besides the differences in the chemical shifts of the aromatic protons caused by complex formation, another question remains - why, for example, is the methine signal H–C(8) of caffeine in coffee so strongly broadened compared to the reference compound and to other signals, like e.g., formic acid? In the following considerations, the methine signal H–C(8) of caffeine is used as an example for the analysis of chemical shifts, line width, and signal intensity of caffeine, as it is the signal within the spectrum that shows no or only negligible overlaps with other signals, in particular those of the melanoidins. Due to the complexity of the coffee matrix and in order to investigate the various influencing factors on the interactions, chemical shifts, and line broadening of caffeine and 5-CQA separately, the system was simplified again. Therefore, equimolar binary model mixtures of caffeine and 5-CQA in different concentrations were compared by ^1^H NMR spectroscopy to the decaffeinated coffee beverage with added caffeine.

To compare the chemical shift differences between these binary solutions and the unaffected single compounds, a computerized addition of the individual ^1^H NMR spectra of 5-CQA and caffeine was performed (Figure 3). With increasing concentrations of the compounds from 0.31 mmol/L up to 5.0 mmol/L a change in the chemical shifts of all aromatic and olefinic signals compared to the artificial computer-aided spectrum of the individual compounds could be observed. Whereas aliphatic signals like H–C(2) - H–C(6) of the quinic acid moiety of 5-CQA were hardly affected. The higher the concentration of caffeine and 5-CQA in the binary equimolar solution, the greater the observed influence on the chemical shift of the aromatic/olefinic protons. A shift toward lower frequencies was observed for all aromatic protons of 5-CQA and caffeine as well as for the methyl groups attached to the purine ring. This is well in line with the observation that the chemical shifts of the individual molecules change when they transition from the free to the complexed state.^41,42^ Sondheimer et al. could observe a strong bathochromic effect, compared to the single compounds, when caffeine and 5-CQA were present in a 1:1 complex in solution.^38^ This supports the assumption that a π-molecular complex was formed, as the higher electron density lowers the excitation energy (π→π*) in the UV spectrum, resulting in a bathochromic shift. In the ^1^H NMR experiment, this increased electron density caused a stronger shielding and consequently a shift to lower frequencies of the protons within the π-molecular complex. These diamagnetic shifts of the ring protons are commonly assigned to the magnetic anisotropy associated with the ring current shielding effect of an aromatic molecule with respect to the opposite aromatic ring in the preferred face-centered stacking (Figure 4). This stacking is favored when electron-rich aromatics (5-CQA) form such a complex with an electron-deficient aromatics (caffeine).^42,43^

**Figure 3:**
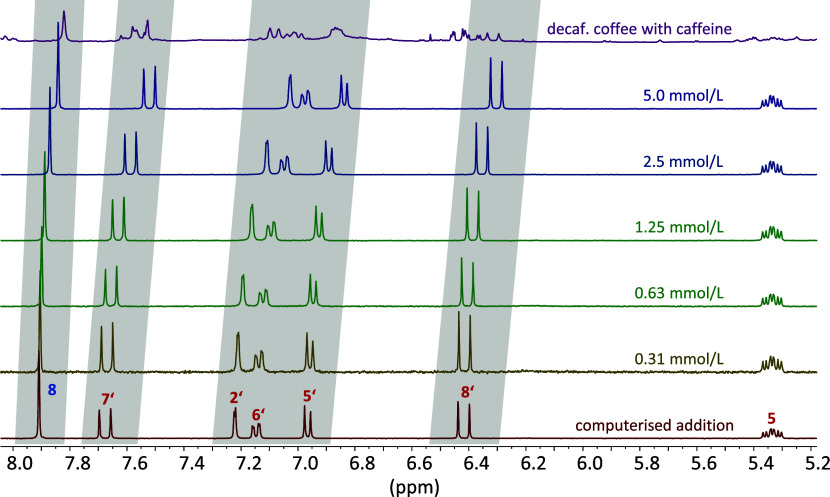
Excerpt of different ^1^H NMR spectra (400 MHz, noesypresat, 540 μL sample, 60 μL NMR coffee buffer, pH = 5.5, 25 °C) of equimolar aqueous solutions of caffeine (0.31 to 5.0 mmol/L) and 5-CQA (0.31 to 5.0 mmol/L) compared to decaffeinated coffee spiked with caffeine (5 mmol/L) and to computer added spectra of the individual compounds (5 mmol/L, each). The numbers of the NMR spectrum at the bottom refer to Figure 2.

**Figure 4:**
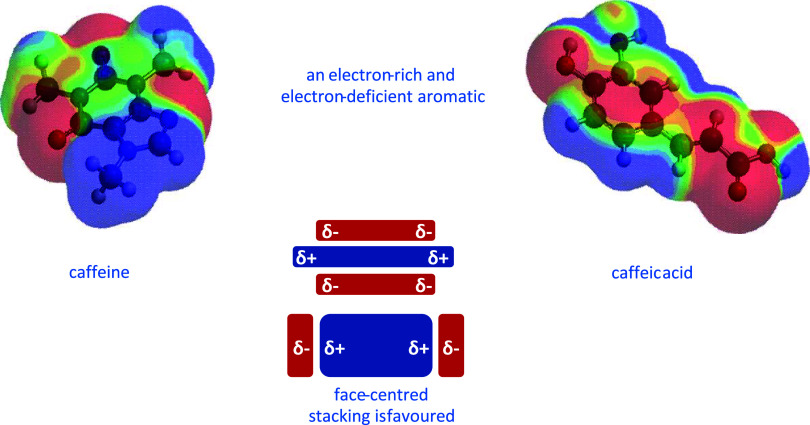
Schemes for describing the electrostatic view of aromatic interactions between caffeine and caffeic acid (in accordance to Martinez et al.).^43^ Calculated by density functional calculations (B3LYP using 6–31G*) with Spartan (Wave function, Inc.).

The observed influence on the chemical shifts of the aliphatic protons of the quinic acid moiety of the 5-CQA was comparatively negligible. In contrast, the aromatic protons H–C(8) (caffeine), H–C­(2′, 5′-8′) (5-CQA) as well as the methyl groups H–C(10–12) of caffeine showed strong differences of the individual chemical shifts depending on the position/orientation of the individual protons, which is well in line with the formation of a π–π molecular complex (Table S1). As an example, H–C(8) of caffeine was shifted by 27 Hz to lower frequencies in the 1:1 (caffeine:5-CQA, 5 mmol/L, each) complex compared to the reference compound (Figure 5A). Self-assembling of the individual compounds was checked, but not significant for the effects observed here (data not shown). Because in literature, the role of the potassium ion was emphasized in the crystalline complex,^38^ it was investigated whether the potassium ion also plays a role in aqueous solution. For this purpose, different concentrations of sodium and potassium chloride were added to the binary solutions with caffeine and 5-CQA, and the chemical shifts were compared to the samples without salt addition. No change in the chemical shifts was observed in any of the samples; regardless of the addition of salt, i.e., in aqueous solutions, cations such as Na^+^ and K^+^ do not seem to have any influence on the complex, in contrast to the crystalline structure (Figure S1).

**Figure 5:**
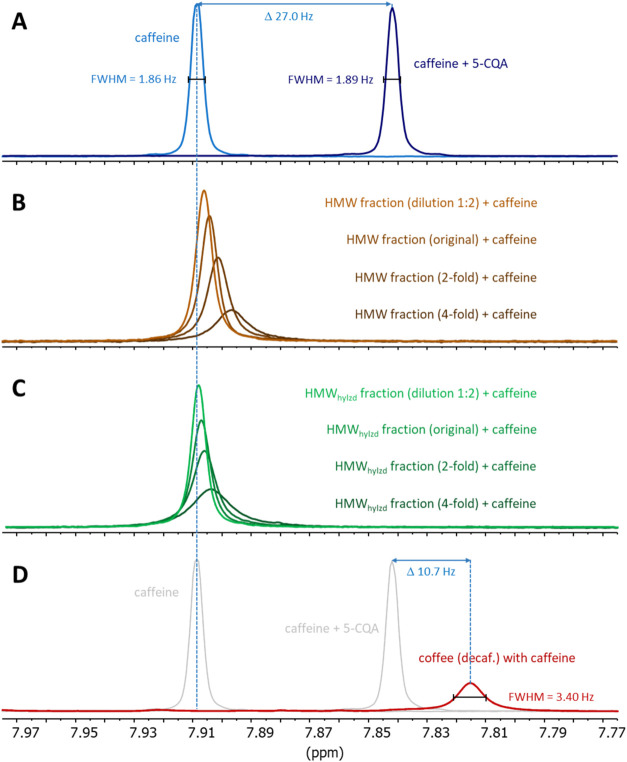
Excerpt of ^1^H NMR spectra (400 MHz, noesypresat, 540 μL sample, 60 μL NMR coffee buffer, pH = 5.5, 25 °C) of caffeine (methine proton, H–C(8)). (A) caffeine (5.0 mmol/L) and caffeine/5-CQA mixture (5 mmol/L, each), (B) HMW fraction: 4- and 2-fold concentrated, original concentration and 1:2 diluted spiked with caffeine (5.0 mmol/L); (C) hydrolyzed HMW fraction: 4- and 2-fold concentrated, original concentration and 1:2 diluted spiked with caffeine (5.0 mmol/L); (D) decaffeinated coffee with caffeine (5.0 mmol/L).

Besides these differences in chemical shifts within the binary complex, the analysis of the line width also revealed huge differences. The methine proton H–C(8) in the coffee beverage matrix showed a strong line broadening of 3.4 Hz compared to the reference or the 1:1 complex with approximately 1.9 Hz and a chemical shift to lower frequencies of additional 10.7 Hz compared to the binary caffeine 5-CQA complex (Figure 5A,D). In contrast to D’Amelio’s work, this study demonstrated that the caffeine-chlorogenic acid complex does not accurately reflect the real coffee system, as neither the peak broadening nor the additional chemical shift of the relevant signals was addressed. However, these observations are consistent with those reported in the literature, which describe line broadening and differences in chemical shift when interactions occur between small molecules and high-molecular-weight compounds.^44,45^ To study the influence of the high molecular weight melanoidins on the complexation-induced changes of the chemical shifts as well as the line broadening,^44,45^ the HMW fraction was isolated from the coffee beverage. The terms “melanoidins” and “HMW” are used interchangeably below based on roasting temperature and time, as it is believed that no unmodified molecules remain in the HMW under these conditions.^27,46^

### Isolation of the High Molecular Weight (HMW) Fraction from a Coffee Beverage

Very recently, Gigl et al. and Gabler et al. showed that the binding activity of odorants strongly depends on the composition of the HMW fraction.^31,48^ For example, interaction studies with furfurylthiol as well as pyrazines before and after alkaline hydrolysis experiments showed a correlation between the release of hydroxycinnamic acids (e.g., caffeic-, ferulic-, *p*-coumaric acid) and the binding affinity of the odorants. Alkaline-treated HMW fractions showed a significantly lower binding affinity to odorants like furfurylthiol or pyrazines compared to the untreated fractions, and this effect could be attributed to π–π interactions. Because caffeine, like pyrazines, is also an electron-deficient aromatic compound, a similar interaction behavior with the HMW fraction was assumed, and therefore, analogous to the pyrazines, NMR-based interaction studies were carried out.^30^ To obtain a pure HMW fraction (>10 kDa) the coffee beverage was separated by ultrafiltration into a high and a low molecular weight fraction. The absence of LMW compounds in the HMW fraction was checked by means of ^1^H NMR spectroscopy. The HMW faction was considered free of LMW if no resonance signals were observable in the region between 5 and 8 ppm and if the intensity of the HMW compounds’ resonances did not change (Figure S2). Finally, the HMW material (>10 kDa) was concentrated, and aliquots were used for the NMR studies as well as the sensory experiments.

Binary solutions of HMW melanoidins in different concentrations and caffeine (5 mmol/L) were prepared and analyzed as described above for the caffeine 5-CQA model mixtures. Compared to protein–ligand interactions, there are only very few examples of interactions between melanoidins and flavor-active molecules (ligands). While protein–ligand based studies are mainly focused on the changes of the NMR signals within the protein, the focus of interactions between melanoidins and flavor active compounds like furfurythiol, pyrazines or caffeine is on the ligand.^30,47,48^ However, the results from protein studies can be applied to melanoidins. In general, the following information can be obtained from the ^1^H NMR spectra: (i) the position of a certain signal within the spectrum provides information about the chemical environment, (ii) the signal area contains information about the concentration of the free ligand in solution and (iii) full width at half-maximum (fwhm) is related to the relaxation behavior of the observed nucleus and is inversely correlated with the transverse relaxation time (*T*_2_). *T*_2_ is related to the molecular weight, the shape of the molecule, the overall correlation time of the molecule, and a possible exchange between different states.^44,49^

As a reference for the evaluation of changes in terms of chemical shifts, line broadening, and signal intensity, caused by interactions of the caffeine with the HMW material, a caffeine solution (5 mmol/L) with and without the addition of high molecular weight material in different concentrations was analyzed (Figure 5A–B). As displayed in Figure 5B a clear trend could be observed. Compared to the reference caffeine solution (Figure 5A), the addition of the HMW fraction caused changes in the chemical shifts, the line broadening, and the peak areas of the selected proton H–C(8). The higher the concentration of the HMW fraction, the more the observed proton is shielded. The NMR signal becomes increasingly broader, and the peak area is significantly reduced up to almost 62% at the maximum for the 4-fold concentrated melanoidin fraction (Figure 5B), compared to the native caffeine reference (Figure 5A). This was determined by quantitative ^1^H NMR spectroscopy (qHNMR). These data clearly demonstrate the presence of molecular interactions between caffeine and the high molecular weight melanoidins in coffee. The possible differences in binding potential between caffeine and the native melanoidins were expressed in the form of free caffeine concentration by qHNMR (Table S2). Previously, a series of experiments was conducted to analyze which hydrolyzable HMW constituents correlate with the observed noncovalent interactions.^31^ Therefore, the major constituents in the HMW hydrolysates, namely monosaccharides, amino acids, organic acids, and hydroxycinnamic acid derivatives, were quantitated via UHPLC-MS/MS. Only the hydroxycinnamic acid content correlated with the interaction activity of the respective HMW fraction. As a result, the present study focused solely on hydroxycinnamic acids, identified as the most significant HMW constituents involved in noncovalent interactions. According to the experiment of Gigl et al., the number of possible binding sites should be reduced after an alkaline hydrolysis, due to the release of interacting melanoidin constituents like caffeic-, ferulic-, *p*-coumaric acid etc.^31^ Therefore, the high molecular weight fraction was hydrolyzed and then again divided into an HMW and an LMW fraction by ultrafiltration. The cleavage of compounds like caffeic-, ferulic-, and *p*-coumaric acid in the LMW fraction was checked by HPLC/UV–vis and quantitated by UHPLC-MS/MS (Table S3) and was well in line with the data published by Gigl et al.^31^ The alkaline hydrolyzed HMW fraction (HMW_hylzd_) obtained in this way was then used for the interaction studies with caffeine. As displayed in Figure 5C, a clear trend could be observed. Analyzing the chemical shift and the intensity of the proton H–C(8) of caffeine clearly showed that in comparison to the untreated HMW fraction (Figure 5B), the hydrolyzed one showed a lower influence on these parameters, which matches well with the fact that possible binding sites were removed during the alkaline hydrolysis.^8^ As knowledge of the structures of coffee melanoidins is still very incomplete, it must be assumed that it is not possible to remove all possible binding sites by hydrolysis. Residual interaction activity observed even in alkaline-treated HMW is most likely due to phenolic or aromatic constituents incorporated in the melanoidins with nonhydrolyzable bonds, such as C–C bonds or ether links. Aromatic amino acids and their Maillard reaction products might also serve as potential interaction partners,^46^ but due to the much higher concentration of phenolic acids (such as caffeic acid), they probably only play minor roles in noncovalent interactions. Therefore, the hydrolyzed melanoidins also continue to interact with caffeine, but in a significantly reduced form. These observations are well in line with similar studies using hydrolyzed melanoidins and odorants like pyrazines or furfurylthiol.^31^

In any scientific approach, model systems such as those used, aim to break down highly complex systems like coffee into appropriate subsystems. Therefore, it can be concluded that the change in the nuclear magnetic resonance spectroscopic properties of caffeine in its pure form compared to the coffee beverage can be well explained by the observed effects. On the one hand, by the formation of the caffeine-chlorogenic acid complex (chemical shift difference) and, on the other hand, by the interactions with the HMW (line broadening) in coffee (Figure 5D).

### Sensory Evaluation of the Caffeine-Chlorogenate and the Caffeine-Melanoidin Complex

The following sensory tests should show whether and to what extent the complex formation affects the taste of caffeine (Table 1). First, the sensory panel evaluated the bitter intensities of an isomolar mixture of caffeine and 5-CQA (7.5 mmol/L, each) at pH 5.5, together with a caffeine reference (7.5 mmol/L, intensity set to 5.0). No significant difference in bitter taste perception could be observed between the reference caffeine solution (intensity 5.0) and the equimolar caffeine-chlorogenic acid complex 4.8 (±0.2). Consequently, the complex seemed to have no or minor influence on the bitter taste of the caffeine or the coffee, respectively. In the next step, the influence of the HMW fraction was evaluated. First, the sensory panel was asked whether the HMW fraction had an intrinsic taste. The panel rated the intrinsic bitter taste of the HMW fraction at only 0.5 (±0.1) compared to the caffeine reference solution (7.5 mmol/L, intensity 5.0). In the subsequent sensory tests, it should be checked to what extent a complex consisting of caffeine, 5-CQA, and the HMW fraction, each in their natural coffee concentrations, affects the bitterness of caffeine. Compared to the caffeine reference (5.0), the solution of the complex was rated with an average of 2.5 (±0.3). Although the HMW fraction showed a small intrinsic bitter taste, the bitterness of the complex consisting of caffeine, 5-CQA, and the HMW fraction was only approximately half as intense compared to the pure caffeine reference solution. The panel also noted a change in the quality of the bitterness, describing it as milder, more coffee-like, and overall, more pleasant. In contrast, the caffeine solution was described as a harsh, alkaloid, medical bitterness. The sensory experiments thus confirm that the interactions between caffeine, 5-CQA, and melanoidins, detected by NMR spectroscopy, significantly reduce the bitter taste impact of caffeine in coffee. In addition to π–π interactions, caffeine may interact with HMW components via hydrophobic interactions and hydrogen bonding, thereby decreasing the concentration of unbound caffeine available to interact with bitter taste receptors. The HMW fraction may also physically obstruct access of caffeine to bitter taste receptors, either by steric hindrance at the receptor surface or by forming a barrier layer, thereby reducing receptor activation and perceived bitterness. To study potential additional effects that may also influence caffeine bitterness in coffee beverages, cell-based taste receptor assays will be conducted in the future.

**Table 1:** Comparison of Sensory Results to Determine the Impact of Complex Formation between Caffeine, 5-CQA, and the HMW Fraction (>10 kDa) of Coffee on Perceived Bitter Taste Intensity

**samples** ^a^	**bitter intensity** ^b^	**significance** ^d^
caffeine (7.5 mmol/L)	5.0 (ref)^c^	
caffeine (7.5 mmol/L) + 5-CQA (7.5 mmol/L)	4.8 (±0.2)	n.s.
HMW > 10 kDa (original coffee concentration)	0.5 (±0.1)	*p* ≤ 0.05
caffeine (7.5 mmol/L) + 5-CQA (7.5 mmol/L) + HMW > 10 kDa (original coffee concentration)	2.5 (±0.3)	*p* ≤ 0.05

a Samples were evaluated in bottled water at pH 5.5.

b Bitter intensities were ranked on a scale from 0 (not detectable) to 5 (strongly detectable). Results are given as the means with standard deviation in parentheses.

c Intensity of caffeine reference (7.5 mmol/L) was set to 5.0.

d Significant differences were determined by *t* test with significance level of α = 0.05, n.s.= not significant.

In summary, it can be concluded that this study was able to demonstrate interactions between the most abundant bitter compound, caffeine, and the high-molecular-weight melanoidin fraction of coffee. Despite simplifying the extremely complex system of coffee into suitable subsystems, it has been possible to answer the question of why coffee does not really taste of caffeine. NMR spectroscopy was used to visualize these interactions and to identify potential interaction partners within the melanoidins, like caffeic acid, ferulic acid, *p*-coumaric acid, etc., by hydrolysis. Furthermore, it was shown that these interactions have a significant influence on the perceived bitterness of caffeine and, consequently, of coffee. Accordingly, a reduced bitterness was noted in the caffeine-5-CQA-HMW-complex, and the quality of the bitterness of the caffeine was also described as less unpleasant, less medicinal, and more coffee-like. In the future, it will certainly be interesting to investigate melanoidins of differently roasted coffees in terms of their interactions with caffeine and the influence on the bitter taste impact. With this knowledge, the sensory perception of instant coffees or other coffee products could be improved by the targeted addition of selected melanoidins.

## Supplementary Material



## Notes

Chemical shifts of caffeine and 5-CQA in different molar ratios; ^1^H NMR spectra of caffeine and CQA with different concentrations of potassium chloride; recovery of caffeine in HMW/HMW_hydlz_ fractions or decaffeinated coffee; ^1^H NMR spectrum of purified HMW fraction >10 kDa; and concentrations of hydroxycinnamic acids after alkaline hydrolysis of HMW (PDF)
