# Supplementary material for: Impact of Interactions between Melanoidins and Caffeine on the Bitter Taste of Coffee Beverages
Source: J Agric Food Chem. 2026 Jun 3;74(23):18243–52. doi: 10.1021/acs.jafc.5c17022 (PMC13281520; doi:10.1021/acs.jafc.5c17022)
Supplement: Supplementary file 1 [file jf5c17022_si_001.pdf]

Supporting Information

The Impact of Interactions Between Melanoidins and Caffeine on the Bitter Taste of Coffee Beverages

Michael Gigl[a,§], Johanna Kreissl[b, §] and Oliver Frank[c,*]

[a]Junior Research Group Food Processing and Health, ZIEL Institute for Food and Health, Technical University of Munich, Lise-Meitner-Str. 34, D-85354 Freising, Germany

[b]Leibniz-Institute for Food Systems Biology at the Technical University of Munich, Lise-Meitner-Straße 34, 85354 Freising, Germany

[c]Chair of Food Chemistry and Molecular Sensory Science, TUM School of Life Sciences, Technical University of Munich, Lise-Meitner-Straße 34, 85354 Freising, Germany

[§]These authors contributed equally.

[*]Corresponding author:

Dr. Oliver Frank
PHONE +49 8161 71 2910
E-MAIL oliver.frank@tum.de

Table of contents

Table S1. Influence on the ^{1}H NMR chemical shifts of caffeine and 5-CQA (400 MHz, 600 µL sample in D2O, 25 °C) at different molar ratios.

Figure S1. Excerpt of different ^{1}H NMR spectra (400 MHz, zg30, 600 µL sample in D2O, 25 °C) of equimolar aqueous solutions of caffeine (2.5 mmol/L) and CQA (2.5 mmol/L) and different concentrations of potassium chloride (1, 10, 100, and 1000 mmol/L), respectively, compared to a mixture without potassium chloride.

Table S2. Recovery of caffeine in mixtures with HMW/HMW_{hydlz} fractions or decaffeinated coffee.

Figure S2. Excerpt of ^{1}H NMR spectrum (500 MHz; H2O/D2O, 9/1, v/v; pH 5.5; 25 °C) of purified HMW fraction >10 kDa isolated from a coffee beverage and monitoring of the purification process (dashed rectangle) by measuring proton NMR spectra after every washing step with 1 liter of water.

Table S3. Concentrations of hydroxycinnamic acids determined after alkaline hydrolysis of HMW material by means of UHPLC-MS/MS to monitor the effectiveness of NaOH treatment.

Table S1. Influence on the ^{1}H NMR chemical shifts of caffeine and 5-CQA (400 MHz, 600 µL sample in D2O, 25 °C) at different molar ratios.

Ratio of 5-CQA:caffeine (v:v)	Shift difference in *Hz* of the respective proton compared to the signal shift in the 50:50 mixture*												
	Caffeine				5-CQA								
	H-C8	H-C10	H-C11	H-C12	H-C2'	H-C5'	H-C6'	H-C7'	H-C8'	H-C2/6	H-C3	H-C4	H-C5
**0:100**	13.9	10.9	10.2	11.0	-	-	-	-	-	-	-	-	-
**5:95**	12.4	9.7	9.2	9.7	-32.6	-21.2	-28.0	-23.8	-15.6	1.96	2.59	1.74	1.78
**10:90**	11.1	8.7	8.1	8.6	-28.9	-19.0	-24.5	-21.3	-13.9	1.82	2.38	1.71	1.49
**20:80**	8.6	6.8	6.4	6.7	-21.8	-14.3	-19.5	-16	-10.3	1.12	1.84	1.31	1.19
**30:70**	5.6	4.4	4.1	4.4	-14.7	-9.8	-13.1	-10.9	-7	0.70	1.24	0.94	0.71
**40:60**	2.7	2.1	2.0	2.1	-7.3	-4.8	-6.2	-5.4	-3.5	0.28	0.65	0.40	0.36
**50:50**	0.0	0.0	0.0	0.0	0.0	0.0	0.0	0.0	0.0	0.0	0.0	0.0	0.0
**60:40**	-2.6	-2.0	-1.9	-1.9	7.4	4.9	6.6	5.6	3.6	-0.56	-0.52	-0.44	-0.46
**70:30**	-5.0	-3.9	-3.8	-3.9	14.8	10.1	13.1	10.9	7.2	-0.93	-1.07	-0.66	-0.85
**80:20**	-7.4	-5.9	-5.6	-5.9	22.1	15.2	19.5	16.7	10.9	-1.31	-1.59	-0.88	-1.05
**90:10**	-9.7	-7.6	-7.3	-7.3	29.8	20.2	26.4	22.3	14.5	-1.68	-2.21	-1.32	-1.57
**95:5**	-11.0	-8.6	-8.2	-7.9	33.4	22.5	29.8	24.9	16.3	-1.87	-2.47	-1.58	-1.83
**100:0**	-	-	-	-	37.2	25.5	33.2	28.1	18.3	-1.87	-2.73	-1.72	-1.89

* „+“ indicates a more deshielded and „-“ indicates a more shielded proton compared to the signal in the equimolar (2.5 mmol/L, each) mixture of 5-CQA and caffeine. The numbers of the individual protons refer to Figure 2.

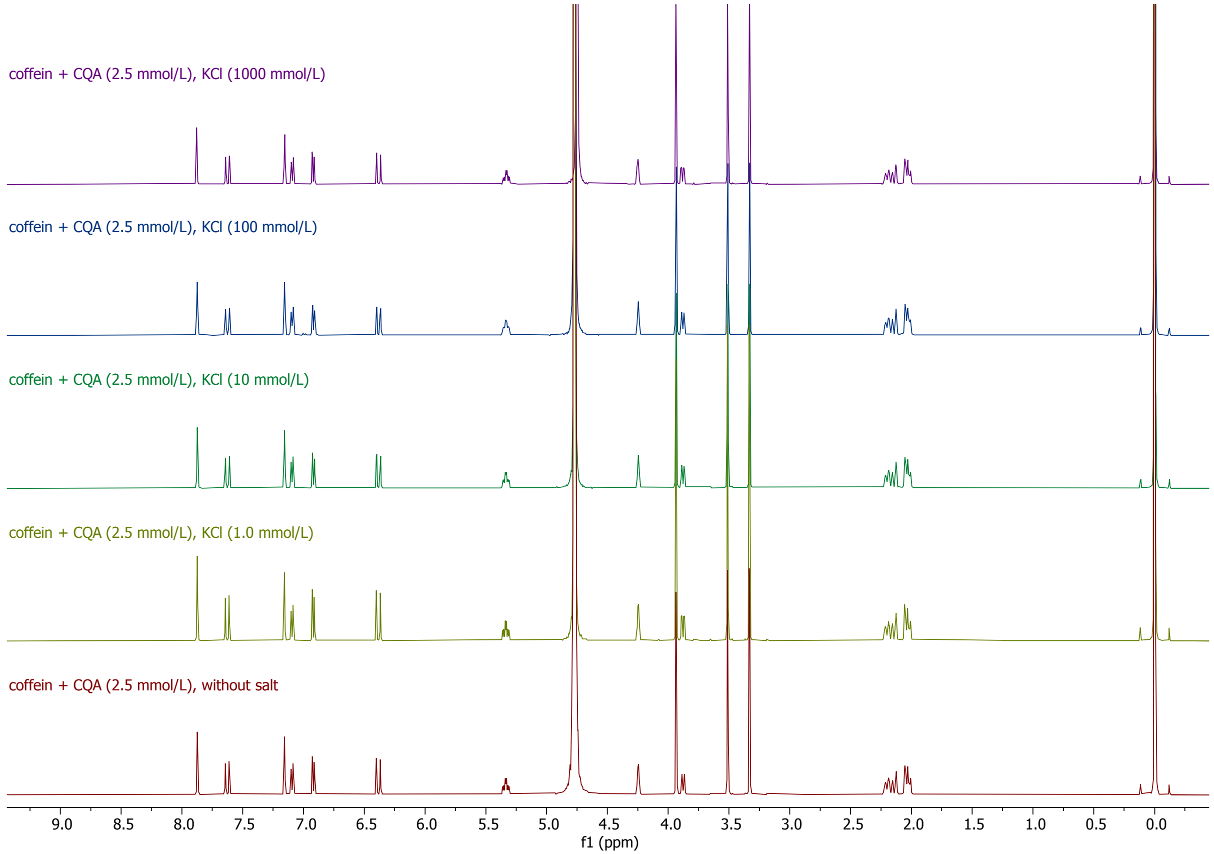

Figure S1. Excerpt of different ^{1}H NMR spectra (400 MHz, 600 µL sample in D_2O, 25 °C) of equimolar aqueous solutions of caffeine (2.5 mmol/L) and CQA (2.5 mmol/L) and different concentrations of potassium chloride (1, 10, 100, and 1000 mmol/L), respectively, compared to a mixture without potassium chloride.

Table S2. Recovery of caffeine (5 mmol/L) in mixtures with HMW/HMW_{hydlz} fractions or decaffeinated coffee.

Dilution of the respective HMW fraction	Caffeine (%) in HMW	Caffeine (%) in HMW_{hydlz}	Caffeine (%) in Decaf*
4:1	38.5	80.0	-
2:1	58.1	83.2	-
1:1	60.7	88.1	-
1:2	76.5	91.1	-
1:4	88.9	94.2	-
1:8	94.2	97.2	-
1:16	95.4	99.6	-
1:32	96.5	100.0	-
Decaffeinated coffee brew	-	-	69.3

* Decaffeinated coffee brew spiked with caffeine (5.0 mmol/L).

Table S3. Concentrations of hydroxycinnamic acids determined after alkaline hydrolysis of HMW material by means of UHPLC-MS/MS to monitor the effectiveness of NaOH treatment.

Hydroxycinnamic acid	Concentration[a] (±SD) (µg/g)
Caffeic acid	8562 (±594.4)
Ferulic acid	685.1 (±42.14)
p-Coumaric acid	146.8 (±17.57)
3,4-Dimethoxycinnamic acid	215.6 (±22.45)

[a]Concentrations were determined in triplicate and expressed as the mean ± standard deviation (SD).

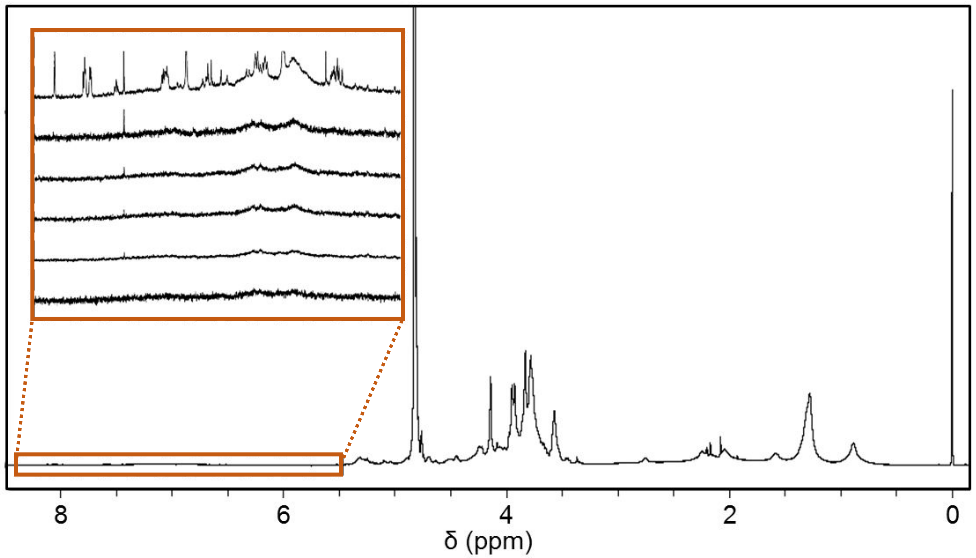

Figure S2. Excerpt of ^{1}H NMR spectrum (500 MHz; H_2O/D_2O, 90/10, v/v; pH 5.5; 25 °C) of purified HMW fraction >10 kDa isolated from a coffee beverage and monitoring of the purification process (orange box) by measuring proton NMR spectra after every washing step with 1 L of water.
